# Targeted Multiple Reaction Monitoring Analysis of CSF Identifies UCHL1 and GPNMB as Candidate Biomarkers for ALS

**DOI:** 10.1007/s12031-019-01411-y

**Published:** 2019-11-12

**Authors:** Shaochun Zhu, Anna Wuolikainen, Junfang Wu, Anders Öhman, Gunnar Wingsle, Thomas Moritz, Peter M. Andersen, Lars Forsgren, Miles Trupp

**Affiliations:** 1 Department of Clinical Science, Neurosciences, Umeå University, Building 10, NUS, Umeå, Sweden; 2 Department of Chemistry, Umeå University, Umeå, Sweden; 3 Department of Forest Genetics and Plant Physiology, Swedish University of Agricultural Sciences, Umeå, Sweden

**Keywords:** CSF biomarker, Proteomics, Parkinson’s disease, ALS, Protein homeostasis

## Abstract

The neurodegenerative diseases amyotrophic lateral sclerosis (ALS) and Parkinson’s disease (PD) share some common molecular deficits including disruption of protein homeostasis leading to disease-specific protein aggregation. While insoluble protein aggregates are the defining pathological confirmation of diagnosis, patient stratification based on early molecular etiologies may identify distinct subgroups within a clinical diagnosis that would respond differently in therapeutic development programs. We are developing targeted multiple reaction monitoring (MRM) mass spectrometry methods to rigorously quantify CSF proteins from known disease genes involved in lysosomal, ubiquitin-proteasomal, and autophagy pathways. Analysis of CSF from 21 PD, 21 ALS, and 25 control patients, rigorously matched for gender, age, and age of sample, revealed significant changes in peptide levels between PD, ALS, and control. In patients with PD, levels of two peptides for chromogranin B (CHGB, secretogranin 1) were significantly reduced. In CSF of patients with ALS, levels of two peptides from ubiquitin carboxy-terminal hydrolase like protein 1 (UCHL1) and one peptide each for glycoprotein non-metastatic melanoma protein B (GPNMB) and cathepsin D (CTSD) were all increased. Analysis of patients with ALS separated into two groups based on length of survival after CSF sampling revealed that the increases in GPNMB and UCHL1 were specific for short-lived ALS patients. While analysis of additional cohorts is required to validate these candidate biomarkers, this study suggests methods for stratification of ALS patients for clinical trials and identifies targets for drug efficacy measurements during therapeutic development.

**Electronic supplementary material:**

The online version of this article (10.1007/s12031-019-01411-y) contains supplementary material, which is available to authorized users.

## Introduction

Amyotrophic lateral sclerosis (ALS) and Parkinson disease are both characterized by progressive neurodegeneration and protein aggregation. The diseases are differentiated by clinical symptoms and upon post-mortem analysis based upon which proteins aggregate in which types of neurons. But, it is an on-going puzzle what protein homeostasis pathways are compromised in each disease and if there is overlap or uniqueness in these molecular deficits. We have sought to use a targeted proteomics platform to characterize the levels in CSF of candidate biomarkers involved in protein degradation pathways. Multiple-reaction monitoring (MRM), using stable isotope-labeled standard (SIS) peptides and liquid chromatography-mass spectrometry (LC-MS)-based separation and identification, allows the quantitative measurement of many proteins simultaneously (Percy et al. 2014; Heywood et al. 2015). MRM method development has been on-going for more than a decade in the area of disease biomarkers in body fluids (Kuhn et al. 2004) and is a promising avenue for quantitative analysis of proteins in translational medicine (Ebhardt et al. 2015; Pal et al. 2014). MRM proteomic analysis of CSF in neurodegenerative diseases has identified some promising candidates in PD and validated biomarkers in Alzheimer’s disease (Heywood et al. 2015; Choi et al. 2013).

Despite the current understanding of the molecular basis of neurodegeneration, extensive drug discovery efforts have not identified any disease modifying medications for neurodegenerative disorders. A key component for successful clinical development programs will be robust diagnostic biomarkers that can differentiate distinct classes within the current clinical diagnosis that may respond differently to drugs. Additionally, such fluid biomarkers may be used as surrogates for clinical responsiveness to medications. Furthermore, a network of biomarkers could function to reveal which molecular deficits a particular medication resolved to increase understanding of disease as well as drive intelligent pharmaceutical development. We have identified disease-related proteins that are detectable by MRM either in our proteomics discovery experiments or as previously reported in the literature. In this study, we have developed a targeted quantitative analysis platform for six proteins involved in neurodegenerative diseases and used it to interrogate their levels in CSF from ALS, Parkinson’s disease, and control patients.

While mitochondrial deficits, oxidative stress, and immune and environmental disruptions are all likely components of neurodegenerative diseases; in this study, we have sought to quantitatively assess the alterations in proteins critical for protein homeostasis in CSF from ALS, Parkinson’s disease, and control patients. We have analyzed samples from a previously published case-control study (Wuolikainen et al. 2016; Wu et al. 2016) that was designed to include rigorously matched patients based on age, gender, and age of sample. We selected a panel of candidate CSF protein biomarkers based on several criteria including previously published proteomics analyses (CHGB); upregulation following lysosomal stress (GPNMB); PD genetics (DJ-1 and GBA); and known mechanisms of protein homeostasis, including proteolysis (CTSD) and ubiquitin-proteasome regulation (UCHL1) (Table 1). We detect a significant decrease in two peptides for CHGB in Parkinson’s disease CSF. In CSF from ALS patients, we measured a significant increase in two peptides from UCHL1 and in GPNMB; these changes were driven by ALS patients that die within 1 year of sampling, as ALS patients that survive longer than 1 year after sampling had levels of these proteins similar to control patients.

**Table 1:** Protein and peptide panel for MRM assays

Gene	Protein	Function	Peptides	Disease relevance
CHGB	Chromogranin B /secretogranin-1	Dense-core vesicle protein	CHGB-GEA, CHGB-NYL	PD proteomics ALS genetics
CTSD	Cathepsin D	Lysosomal protease	CTSD-VST, CTSD-YSQ	Increased in ALS spinal cord
PARK7	Protein DJ-1	Protease, glycase	DJ1-EGP, DJ1-GPG	PD genetics
GBA	Glucosylceramidase	Lysosomal hydrolase	GBA-NFV, GBA-SYF	PD genetics
GPNMB	Transmembrane glycoprotein NMB	Regulation of protein degradation and inflammatory response	GPNMB-AYV	PD genetics
UCHL1	Ubiquitin C-terminal hydrolase L1	Ubiquitin-proteasome dual hydrolase-ligase function Maintenance of axonal structural integrity	UCHL1-LGF, UCHL1-LGV	PD-like syndrome with corticospinal motor neuron loss

## Materials and Methods

### Experimental Design and Statistical Rationale

This study continues the molecular characterization of a carefully selected cohort of matched sets of Parkinson’s disease, ALS, and control patient samples chosen to reduce differences in the age of patient and age of sample (Wuolikainen et al. 2016; Wu et al. 2016). From biobanks at the University Hospital in Umeå, rigorous matching of ALS, PD, and control samples yielded 21 ALS, 21 Parkinson’s disease, and 25 control patient samples for the current analysis. Control samples were collected from patients with other neurological symptoms unrelated to ALS or Parkinson’s disease. For a detailed description of individual patient matches for age of patient and sample, please refer to Supplementary Table 1 in (Wuolikainen et al. 2016). Run order was determined using constrained randomization (Jonsson et al. 2015) to maintain matched groups of patients and controls to be run next to each other, but randomized within groups (Supplemental Table 2). Briefly, CSF samples were collected from non-fasting patients visiting the Neurology ward at Umeå University Hospital and stored in − 80° freezers. Confident diagnoses were obtained during follow-up visits from multiple neurologists expert in neurodegenerative diseases, and confirmed in many cases by post-mortem analysis. The Parkinson’s disease patients were investigated according to the UK PDSBB criteria, and the ALS patients were investigated according to the revised European Federation of Neurological Societies Consensus Guidelines for Managing ALS (EFNS task force on diagnosis and management of ALS 2012). This study was performed in accordance with the Declaration of Helsinki (World Medical Association 2013) and was approved by the Swedish medical ethical review board. Written informed consent was obtained from all subjects.

### Statistical Analysis

Each sample was spiked with 11 SISs and integrations, and ratio of relative response was performed using Skyline and exported for further analysis. Standard curves were used to calculate concentrations for each peptide and the concentration of the target protein. Most univariate analyses were performed on R software (version 3.2.2); and receiver operating characteristic (ROC) curves were done using the web-based online tool MetaboAnalyst (https://www.metaboanalyst.ca). ROC curve optimum cutoff value was defined as the value associated with the maximal sum of sensitivity and specificity.

Multivariate data analysis was performed using SIMCA software (version 13.0.3, Umetrics, Umeå, Sweden). UV (unit variance scaling) was used for data scaling. Principal components analysis (PCA) was applied for overview of sample distributions among groups and for detection of outliers (Wold et al. 1987). Orthogonal partial least squares discriminant analysis (OPLS-DA) was used to detect peptide patterns that best discriminate between the pre-defined sample groups with *R*^2^ and *Q*^2^ for indication of the model performance and predictive ability (Trygg and Wold 2002). In addition, *p* values were calculated for each OPLS-DA model using analysis of variance (ANOVA) (Eriksson et al. 2008). OPLS-DA models were optimized by iterative testing for peptides to identify the most significant multivariate models.

### Protein Panel and Peptide Synthesis

The criteria of peptide sequences chosen include sequence uniqueness, absence of internal tryptic cleavage sites, and absence of amino acid residues with a high propensity for spontaneous modifications such as methionine. As a result, 11 peptides are chosen as shown in Table 1. Proteins for targeted analysis were selected from known Parkinson’s disease genes that were detected by LC-MS analysis of fractionated CSF in our laboratory (not shown) or others, or in previous untargeted proteomics experiments. Names of peptides are constructed using “gene name abbreviation + the first three N-terminal amino acids of the peptide.” The peptides were purchased from Cambridge Research Biochemicals and exhibit purity > 95% (*via* reversed-phase (RP) LC and mass determined by matrix-assisted laser desorption/ionization time-of-flight mass spectrometry); the net peptide content was determined by elemental analysis (C, H, N).

### Sample Preparation

Pooled CSF for quality control was made by pooling aliquots from all of the CSF samples. Protein content was measured by Coomassie (Bradford) Protein Assay Kit (Part No.23200, Thermo) for each sample. Fifty-microliter individual sample or 100-μL pool aliquots were trypsin digested. Samples were reduced with equal volume of reducing buffer (10 mM tris (2-carboxyethyl) phosphine, 2% sodium deoxycholate, 50 mM ammonium bicarbonate) for 30 min at 60 °C. Then, the reduced disulfides were alkylated with final 10 mM iodoacetamide through 30-min incubations in the dark at room temperature. Digestion was performed using sequencing grade modified trypsin (catalog V5111 Promega) spiked in at a 50:1 protein/enzyme ratio. After 18 h at 37 °C proteolysis was stopped by addition of chilled formic acid (FA) solution (0.5% final).

After pelleting the acid insoluble surfactant by centrifugation (14,000*g* for 10 min), supernatant was transferred to clean tubes and mixed with stable isotope standard (SIS) peptides (concentration balanced to match the endogenous (native, NAT) peptide in the protein quantitative analyses). For quantification curves, 8 SIS peptide concentrations (A1-A8) spanning a 2187-fold range (from 50 to 0.023 fmol/μL) were prepared at a ratio of 1:3:3:3:3:3:3:3. Peptide mixtures were desalted on 10 mg Oasis HLB cartridges (part no. 186000383; Waters; Milford, MA, USA). The eluent (65% acetonitrile (ACN), 0.1% FA) was then dried in a speedvac (SP Scientific) at RT for 3 h, the sample rehydrated with 0.1% FA (50 μL for individual sample or 100 μL for pool aliquots) followed immediately by (RP)-ultra high-performance (UHP)LC-MS analysis.

### RP-UHPLC/MS Conditions

Twenty microliters CSF digests (4–10 μg) were loaded and separated by Aeris XB-C18 (150 × 2 mm, 2.6 μm particles; part no. 00F-4505-AN; Phenomenex) protected with a guard column (SecurityGuard ULTRA Cartridges, part no. AJ0-8948, Phenomenex) at 0.5 mL/min over a 35-min ACN gradient. The gradient was modified from Percy AJ et al. (Percy et al. 2012) as follows (time, %B): 0, 1; 0.1, 10; 3, 11; 13, 19; 13.5, 20; 13.6, 23; 16.7, 25; 19.7, 28.5; 21.7, 34; 22.5, 42; 23.5, 90; 29, 90; 30, 1; and 35, 1. The composition of the mobile phases was 0.1% FA in water for A and 0.1% FA in 90% ACN for B. A 1290 Infinity system (Agilent Technologies, Waldbronn, Germany) was used with the column and autosampler maintained at 40 and 4 °C, respectively.

The peptides were detected with an Agilent 6490 triple quadrupole (QqQ) mass spectrometer equipped with a jet stream electrospray source operating in positive ion mode. The jet-stream gas temperature was 150 °C with a gas flow of 16 L/h, and a sheath gas temperature to 350 °C and flow rate of 11 L/h; nebulizer pressure was set to 35 psi. The capillary voltage was set at 4 kV, and nozzle voltage was set to 300 V. The fragmentor voltage was 380 V, and 6 V was used for cell accelerator potential. The MS was operated in dynamic MRM mode, with unit resolution (0.7 Da full width at half maximum, i.e., FWHM) in the first quadrupole (Q1) and the third quadrupole (Q3). Delta retention time was 4 min and the cycle time was 600 ms.

### MRM Transition Selection and Parameter Optimization

All possible transitions were experimentally tested in the Agilent 6490 mass spectrometer. Optimized collision energy (CE) voltages were estimated by Skyline software (version 3.1, MacCoss Lab, University of Washington) and optimized through increasing voltage steps of 1 V from − 5 V to + 5 V around the predicted CEs. Since SIS peptides have the same behaviors as their native (NAT) peptide in separation, ionization, and fragmentation, the same MRM acquisition parameters and retention times were used for both peptide forms. The only difference was the actual precursor and product ion m/z values. The top five most intense ions were chosen from each SIS peptide in buffer. SISs were also spiked in CSF to monitor both SIS and NAT behaviors. Only transitions that show no interference in CSF, the most response, and least variation (≤ 20% coefficient of variation (CV) between its ratios) were selected, and three transitions were chosen for each peptide for monitoring in the final assays.

### Metabolomics

Metabolite analysis methods are completely described in previously published studies using MS (Wuolikainen et al. 2016) and NMR (Wu et al. 2016).

### Data Analysis

All data files were imported into Skyline (version 3.1) for peak integration with manual validation. Peak specificity between the endogenous and SIS MRM signal was defined as the detection of at least one transition from the endogenous peptide exactly co-eluting with at least two transitions from the stable isotope-labeled peptide and also with dot-product ratios > 0.94. Linear regression was used to fit the serial dilution data points for each curve (weights = 1/*x*^2^). Precision was calculated for each level of the standard curves using CV (the standard deviation (SD) divided by the mean, expressed as a percentage). Limit of detection (LOD) was determined by 3 × *s*_y|x_/slope (*s*_y|x,_ standard error of y-estimate in the regression equation) (Anderson 1989). Lower limits of quantification (LLOQ) were calculated as three times LOD if CV of the lowest calibration levels of standard curves was below 20%. Otherwise, LLOQ was determined by the lowest calibration level with CV less than 20%. An assay was specific when at least one light transition co-eluted with at least two heavy transitions. Assays were considered successful when they were precise and specific. Protein concentrations were calculated by using the following formula: protein concentration (ng/mL) = concentration of peptide (fmol/μl)/1000 × MW (molecular weight) of the target protein (from the UniProtKB database).

## Results

### Development of Multiplexed Assays

For candidate peptide validation, we used 11 peptides covering six different proteins previously shown to be altered in Parkinson’s disease (Table 1). Each peptide was selected based on uniqueness to the target protein. The best collision energies (CEs) were determined for each peptide by experimentation. Three transitions per peptide were identified with highest specificity and sensitivity for CSF measurements as shown in Supplementary Table 1. Eleven peptides, representing six proteins were detected and validated in CSF assays (Supplemental Table 1), five of these proteins have two peptides including chromogranin B/secretogranin-1 (CHGB), cathepsin D (CTSD), ubiquitin carboxyl-terminal hydrolase isozyme L1 (UCHL1/PGP9.5), protein DJ-1, and glucosylceramidase (GBA), while transmembrane glycoprotein NMB (GPNMB) was quantified using a single peptide.

Standard curves were used to evaluate the linear relationship between response and concentration of spiked stable-isotope labeled standard (SIS) and to calculate concentrations of targets in samples. Here, eight concentrations spanning over 2000-fold range (from 50 to 0.023 fmol/μL) were tested for all peptides. All standard curves had good correlation between relative responses of SIS to NAT and SIS concentration with CVs calculated from triplicate measurements for each level of peptides (Supplementary Fig. 1 and Supplementary Table 2). Two peptides, GBA-SYF and GBA-NFV, had high CVs (6.90–31.9% for GBA-SYF, 5.30–36.7% for GBA-NFV) across all levels of standard curves; this is the result of NAT levels below the lower limit of quantification (LLOQ) using the current analytical setup. However, these peptides were detected with almost all transitions and eluted from the LC column exactly with the SIS (Supplementary Fig. 2), so were retained for determination in subsequent CSF measurements. Concentration of these two peptides were calculated by (NAT/SIS) *C_SIS_ (NAT/SIS, ratio of response of NAT to SIS, C_SIS_ was concentration of SIS spiked in each sample). Except for these two peptides, concentration of other peptides was calculated by their own standard curves. LOD could be detected at lower than 0.086 fmol/μL. Accounting for molecular weight of each protein, most proteins could be quantified below 1.71 ng/ml as shown in Supplementary Table 2 and the LLOQ of protein CTSD was as low as 0.50 ng/ml.

### Multiplexed Analysis of Targeted Proteins in CSF

In this study, we analyzed CSF samples from 21 ALS, 21 PD, and 25 control patients from a previously described cohort rigorously selected and matched for age of patient, age of sample, and gender (Wuolikainen et al. 2016; Wu et al. 2016). Univariate analysis as depicted in Table 2 indicates that both peptides from CHGB (ratios = 0.68) were reduced in PD compared to control with high statistical significance. Also, UCHL1-LGF (ratio = 0.83, *p* = 0.003) and GBA-SYF (ratio = 0.82, *p* = 0.031) were decreased in PD compared to controls. In ALS, both peptides from UCHL1 were increased in CSF compared to control (UCHL1-LGF: ratio = 1.22, *p* = 0.005, UCHL1-LGV: ratio = 1.84, *p* = 0.002) and GPNMB levels were also increased in ALS compared to controls (ratio = 1.37, *p* = 0.020). In addition, one peptide from CTSD was also found to be higher in ALS than in controls (ratio = 1.13, *p* = 0.009).

**Table 2:** Univariate analysis of peptide levels compared between ALS, PD, and control samples

Peptides	Mean (ng/ml)			ALS/control		PD/control		PD/ALS	
	Control	ALS	PD	Ratio	*p* value*	Ratio	*p* value	Ratio	*p* value
CHGB-GEA	1213	1019	826	0.84	0.124	0.68	0.0012*	0.81	0.083
CHGB-NYL	653.1	570.7	441.0	0.87	0.213	0.68	0.00037*	0.77	0.025*
CTSD-VST	61.88	69.74	63.10	1.13	0.009*	1.02	0.701	0.90	0.016*
CTSD-YSQ^a^	54.35	58.13	54.38	1.07	0.497	1.00	0.996	0.94	0.523
DJ1-EGP	10.44	10.16	9.63	0.97	0.787	0.92	0.407	0.95	0.616
DJ1-GPG	7.15	6.72	6.52	0.94	0.251	0.91	0.094	0.97	0.584
GBA-NFV	2.47	2.43	2.09	0.98	0.829	0.84	0.070	0.86	0.086
GBA-SYF	4.69	4.56	3.84	0.97	0.756	0.82	0.031*	0.84	0.113
GPNMB-AYV	7.65	10.52	6.27	1.37	0.020*	0.82	0.096	0.60	0.002*
UCHL1-LGF	0.96	1.16	0.79	1.22	0.005*	0.83	0.003*	0.68	5.4E−6*
UCHL1-LGV	0.91	1.68	0.74	1.84	0.002*	0.81	0.182	0.44	0.001*

Ratio is mean values of group 1/group 2

*Significant by Student’s *t* test with *p* value < 0.05

^a^Removed from further statistical analysis due to genetic variation skewing peptide results

**Fig. 1:**
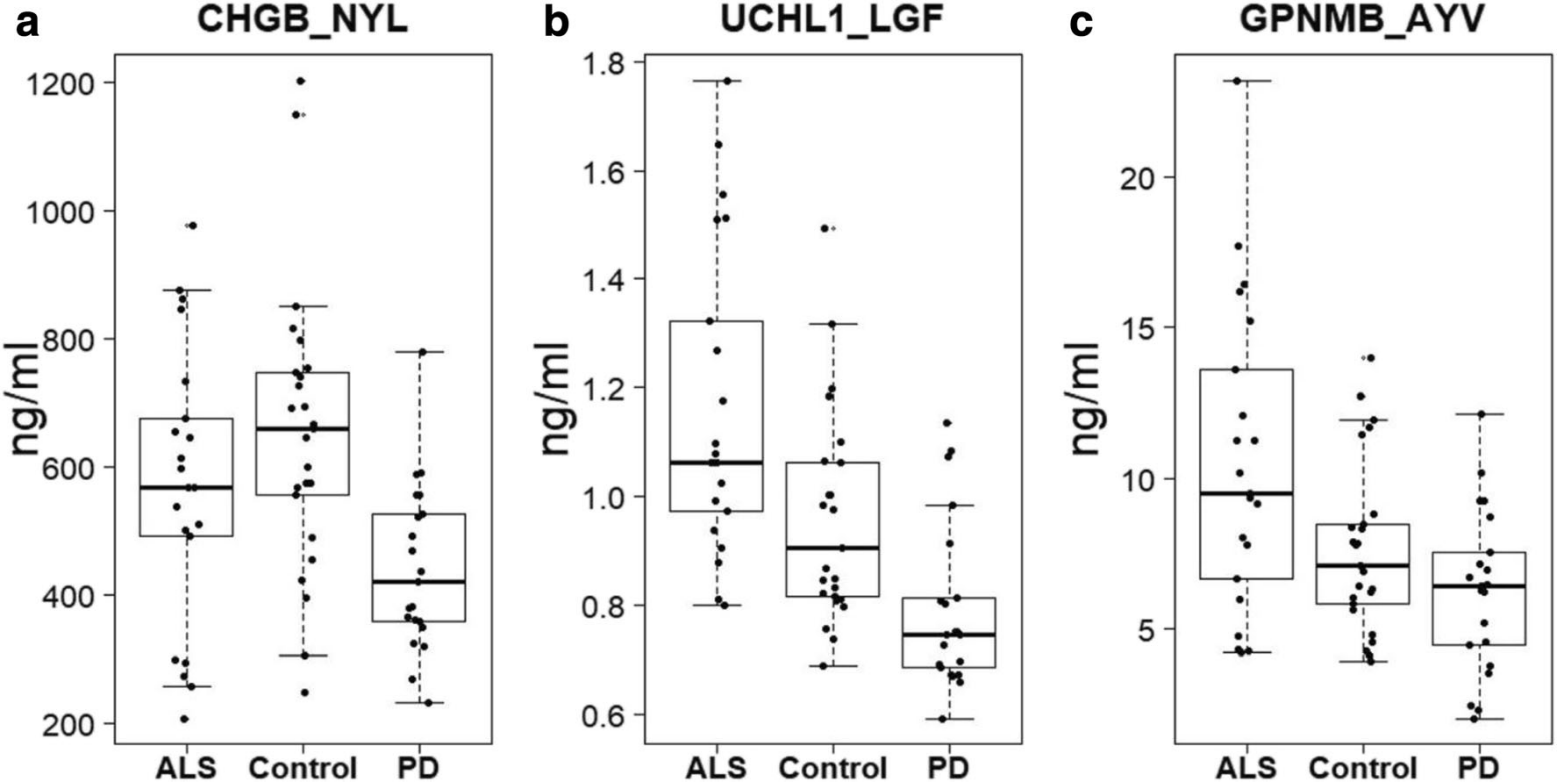
Boxplots of peptides most significantly different between control and patients with PD or ALS. Peptides were selected based on most significant difference between control and patients with Parkinson’s disease (**a**), or control and ALS patients (**b**, **c**). **a** CHGB_NYL. **b** UCHL1_LGF. **c** GPNMB_AYV. Concentrations (ng/mL)

The distribution of peptide measurements within disease groups is represented by the box plots for the most significant proteins in PD and ALS vs control in Fig. 1. There is considerable spread amongst measurements for the control group, as may be expected amongst a diverse group of disease controls, but clear trends can be observed compared to PD for CHGB-NYL (Fig. 1a). For ALS, there is a broad range of measurements for the peptides depicted. Therefore, we sought to characterize the peptide measurements in ALS patients stratified based on longevity following date of CSF sampling to determine if the spread could originate from differences in disease progression.

### Targeted Peptide Analysis of Longevity Differences in Patients with ALS

While the 21 patients in this study were selected based on other criteria, they are randomly distributed into nearly equal groups based on survival following CSF sampling: 10 patients died within 1 year, while 11 patients survived more than 500 days. By univariate analysis as shown in Table 3, three peptides from two proteins (UCHL1 and GPNMB) are significantly increased in short-lived ALS patients (ALS.S) compared to longer-lived ALS patients (ALS.L). While these increases are also seen in ALS.S compared to controls, the levels of these proteins are not significantly different between ALS.L and control.

**Table 3:** Most significant peptides differentiating between control and short- and long-lived ALS

Peptides	Mean (ng/ml)			ALS.S/ALS.L		ALS.S/control		ALS.L/control	
	Control	ALS.S	ALS.L	Ratio	*p* value	Ratio	*p* value	Ratio	*p* value
CHGB_GEA	1213	963	1070	0.90	0.554	0.79	0.116	0.88	0.372
CHGB_NYL	653.1	535.3	603.0	0.89	0.493	0.82	0.154	0.92	0.546
CTSD_VST	61.88	70.41	69.14	1.02	0.699	1.14	0.034*	1.12	0.067
GPNMB_AYV	7.65	13.22	8.08	1.64	0.016*	1.73	0.00027*	1.06	0.705
UCHL1_LGF	0.96	1.30	1.04	1.25	0.032*	1.36	0.0003*	1.09	0.257
UCHL1_LGV	0.91	2.24	1.17	1.90	0.019*	2.45	1.5e-5*	1.29	0.166

ALS.S are ALS patients with short survival; ALS.L are ALS patients with long survival

*Significant by Student’s *t* test with *p* value < 0.05

**Fig. 2:**
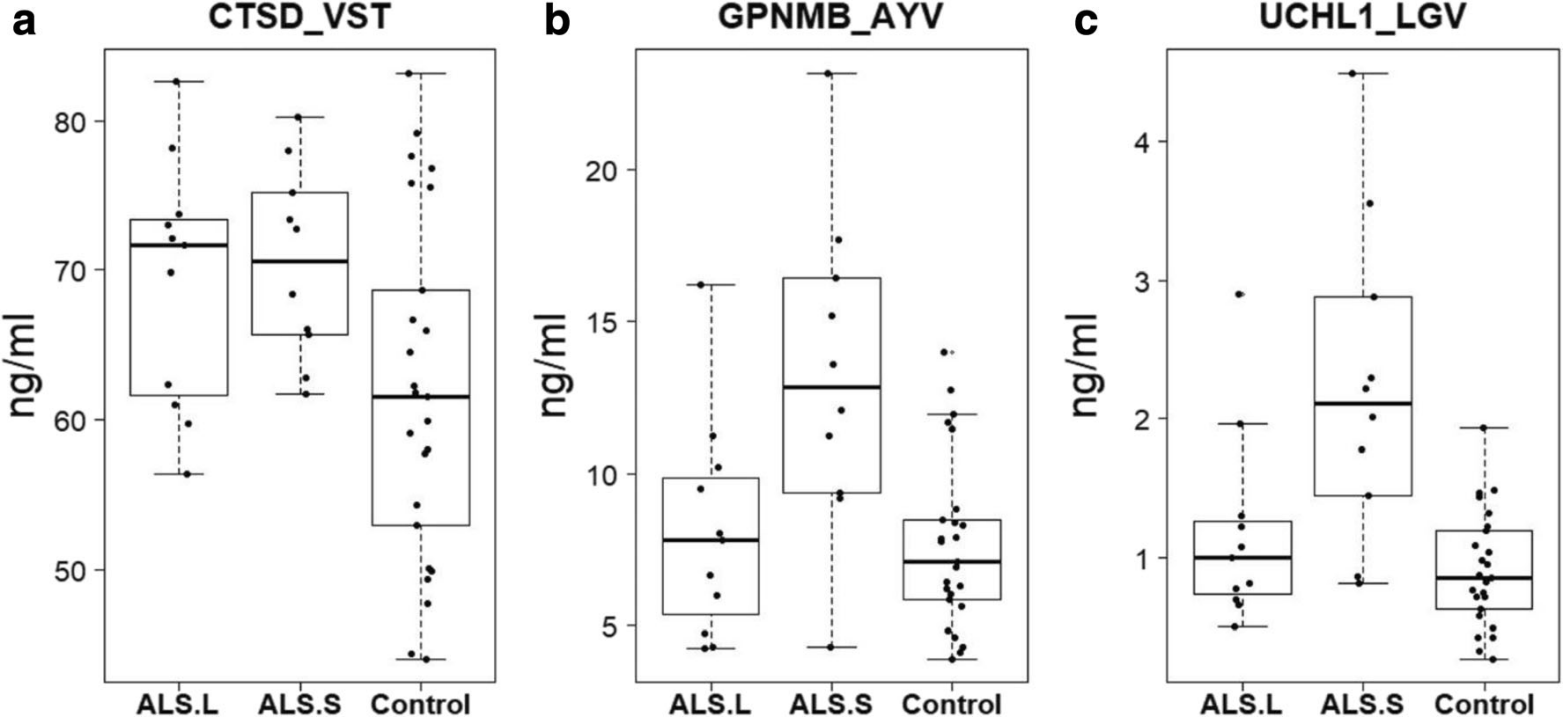
Boxplots of peptides most different between control and long- or short-lived ALS. **a** CTSD_VST. **b** GPNMB_AYV. **c** UCHL1_LGV. Concentrations (ng/mL). ALS.S ALS patients with shorter survival, ALS.L ALS patients with longer survival

The box plots comparing the distribution of individual patients in the ALS-S, ALS.L, and control groups depicted in Fig. 2 reveal that levels of CTSD are not significantly different between ALS.S and ALS.L, but they are significantly increased in ALS.S compared to control due to the tighter clustering of this cohort of ALS.S patients compared to ALS.L (Fig. 2a). While there are some outliers amongst ALS.L with higher levels of GPNMB and UCHL1, ALS.L patients have levels of these peptides similar to control samples (Fig. 2b, c). The ALS.S patients also exhibit a broad range of levels of these peptides, but the boxed values (the middle 2 quartiles centered around the median) are above and clearly separated from the similarly boxed values for the control patients (Fig. 2b, c).

### Multivariate Analysis of MRM Measurements

**Fig. 3:**
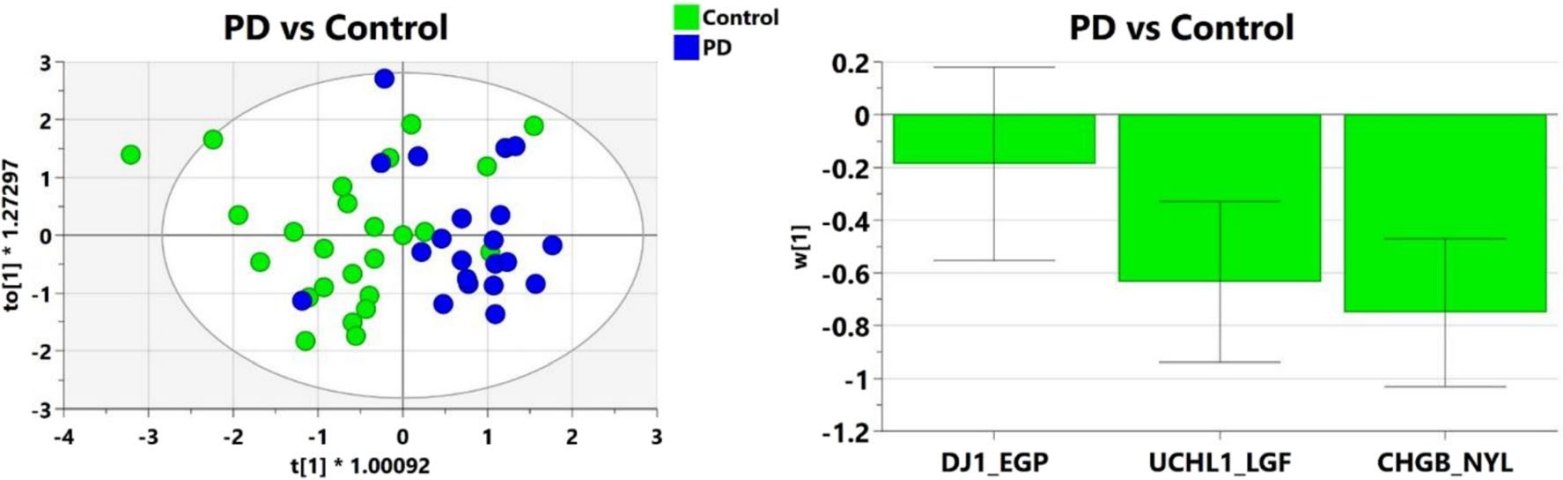
Multivariate analysis of proteins different between Parkinson’s disease and control. Peptides were selected based on optimization of the OPLS-DA model. Left, scores plot of two component OPLS-DA model, R2X = 0.81, R2Y = 0.367, Q2 = 0.297, *p* = 0.0051; right, histogram of model weights for each peptide in model (w plot).

Orthogonal projection to latent structures discriminant analysis (OPLS-DA) was used to detect peptide patterns that best discriminate between the disease groups and controls. Initial OPLS-DA models were built using all measured peptides (not shown) and then optimized for highest statistical significance by selecting peptides of higher absolute value of w* than related w*cvSE. Initial modeling of PD vs Control using all measured peptides was significant (not shown; Q2 = 0.0.171, *p* = 0.018) but refinement using the combination of CHGB-NYL, UCHL1-LGF, and DJ1_EGP resulted in an optimized model (Fig. 3; Q2 = 0.297, *p* = .0051). But with several control patients showing overlap with PD patients, additional peptides are needed for better classification in future analyses.

**Fig. 4:**
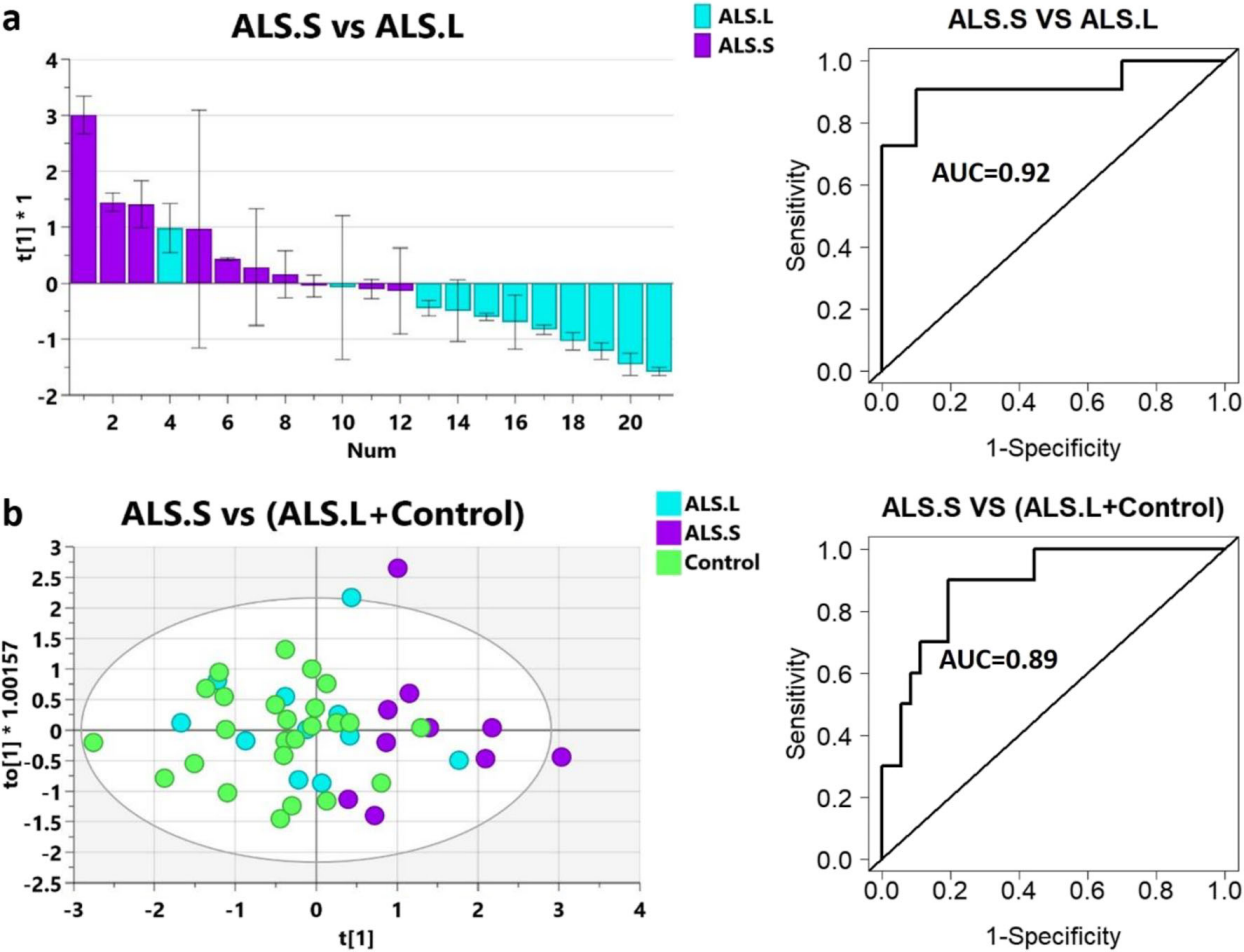
Modeling analysis of ALS short-lived compared to ALS long-lived and control using peptides UCHL1_LGV and GPNMB_AYV. Models were optimized by iterative testing for peptides resulting in the most significant OPLS-DA model for ALS.S vs ALS.L. **a** Separation of patients with ALS based on longevity, ALS.S vs ALS.L. Left, scores plot of single component OPLS-DA model, R2X = 0.611, R2Y = 0.431, Q2 = 0.313, *p* = 0.034; right, ROC analysis was done using log2(UCHL1_LGV*GPNMB_AYV). **b** OPLS-DA model of short-lived ALS vs control + ALS.L. Left, scores plot of individual patient measurements, model statistics: R2X = 1, R2Y = 0.416, Q2 = 0.324, *p* = 0.0025; right, ROC analysis was done using log2(UCHL1_LGV*GPNMB_AYV)

The optimized OPLS-DA model of the ALS.S group versus ALS.L samples was obtained by using two peptides (UCHL1-LGV and GPNMB_AYV). The refined model showed improved classification between the groups defined by longevity (Fig. 4a; Q2 = 0.313, *p* = 0.034). These two peptides were used in multivariate modeling to distinguish between short-lived ALS patients and all other samples. Good classification was observed in an OPLS-DA model between ALS.S and Control+ALS.L (Fig. 4b; Q2 = 0.324, *p* = 0.0025).

To further assess the diagnostic potential of these two peptides, receiver operating characteristic (ROC) curves were generated using the concentrations of proteins based on the UCHL1_LGV and GPNMB_AYV peptides. Area under the curve (AUC) values from ROC analysis were calculated and suggest that MRM measurements using these two peptides may provide a robust diagnostic tool for differentiating short-lived ALS patients from longer-lived ALS patients (Fig. 4a, AUC 0.92) or from within a combined sampling of ALS and control CSF (Fig. 4b, AUC 0.89).

### Metabolomics Profiles and ALS Longevity

**Fig. 5:**
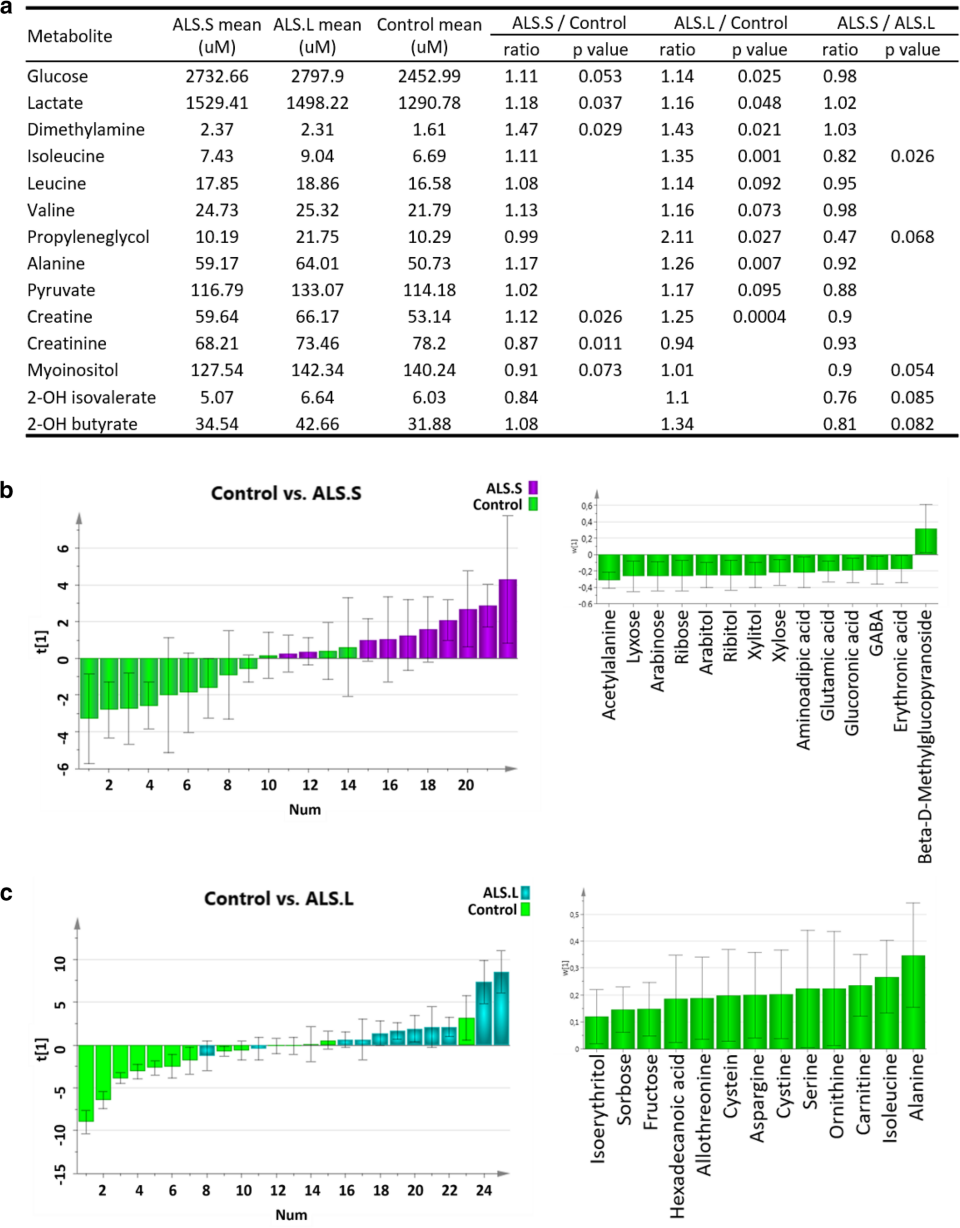
Analysis of CSF metabolite differences between short- and long-lived ALS patients. **a** Univariate analysis of NMR metabolite levels in ALS short-lived (ALS.S), ALS long-lived (ALS.L), and control patients. **b** Multivariate modeling of mass-spectrometry metabolomics measurements for short-lived ALS patients versus control. Left, scores plot of single component OPLS-DA model; right, histogram of model weights of most important metabolites for separating ALS.S from controls. **c** Multivariate modeling of mass-spectrometry metabolomics measurements for long-lived ALS patients versus control. Left, scores plot of single component OPLS-DA model; right, model weights of metabolites separating ALS.L from controls

Due to the small size of short- and long-lived subsets of ALS patients, we sought to validate differences in these subgroups by analyzing data from additional analytical methods. Thus, we have studied the previously published metabolomic profiles from the same CSF samples with regard to longevity of ALS patients. In earlier multi-platform metabolomics analyses (Wuolikainen et al. 2016; Wu et al. 2016), we detected increased levels of branched chain amino acids; here for the first time, dividing ALS patients based on longevity reveals that the increase in isoleucine by NMR measurements (Fig. 5a) is only significant for long-lived ALS patients (ratio 1.35, *p* = 0.001). Isoleucine is increased in long- versus short-lived patients (ratio 1.22, *p* = 0.026). Also, increases in valine and leucine levels were borderline significant (*p* < 0.1) by NMR only for long-lived ALS patients (Fig. 5a). ALS patients with a longer post-sampling life span also show increased CSF levels of propylene glycol (ratio 2.11, *p* = 0.027) (Fig. 5a). The shorter longevity ALS patients were the only subgroup to show a significant decrease in creatinine compared to control (ratio 0.87, *p* = 0.011). Myoinositol was also lower in short- compared to long-lived ALS (ratio 0.9, *p* = 0.054). The increases in ALS CSF measurements by NMR were more pronounced in long- than short-lived patients compared to control for creatine (ratio 1.25, *p* = 0.0004; ratio 1.12, *p* = 0.026, respectively) and alanine (ratio 1.26, *p* = 0.007; ratio 1.17 *p* = 0.13). In contrast, glucose, lactate, and dimethylamine were altered similarly in long- and short-lived ALS patients (Fig. 5a). Two metabolites that exhibited borderline significant differences between short- and long-lived ALS patients warranting further targeted analysis in larger cohorts are 2-hydroxyisovalerate and 2-hydroxybutyrate (Fig. 5a).

We have also detected increases in isoleucine and alanine only in long-lived ALS patients in mass-spectrometry-based metabolomics measurements. Multivariate analysis reveals that increases in alanine (weight in model, w* 0.348), isoleucine (w* 0.267), and carnitine (w* 0.236) are important for separating long-lived ALS patients from control in OPLS-DA models (Fig. 5c). Short-lived ALS patients were most significantly different from controls in variable selected OPLS-DA models of MS measurements due to decreases in the 5-carbon sugars arabinose (w* − 0.265), ribose (w* − 0.260), and xylose (w* − 0.219), and their alcohol derivatives arabitol (w* − 0.252), ribitol (w* − 0.251), and xylitol (w* − 0.251) (Fig. 5b). Additionally, from MS metabolite measurements, decreased acetylalanine (w* − 0.313) and aminoadipic acid (w* − 0.218) and increased beta-d-methylglucopyranoside (w* 0.317) were important in multivariate models separating short-lived ALS from controls (Fig. 5c).

## Discussion

We have sought to assay a diverse range of molecular functions by targeted quantitative analysis of six proteins involved in protein homeostasis. Such a panel of biomarkers will have two main uses for clinical development of therapeutics: to identify subgroups within a broad clinical diagnosis that can be selected for enrollment in trials for targeted therapies; and as a diagnostic marker for remediation of disease alterations during a course of treatment. The MS-based MRM assays described here are robust, multiplexed, and sensitive enough to function as companion diagnostic biomarker tools.

### Candidate Peptide Biomarkers for PD and ALS

Here, we have shown a decrease in PD CSF levels of two peptides from the chromogranin B protein (CHGB), also called secretogranin 1. CHGB may function as a marker for alterations in the regulated secretory pathway in neurons. Decreases may be a result of neuronal loss due to disease progression or may signify decreased protein secretion from intact neurons, possibly including proteins prone to misfolding such as APP fragments (Mattsson et al. 2010). CHGB is involved in secretory vesicle formation and regulation and has been shown altered in previous studies of Parkinson’s disease, including an interesting model of axonal dynamics (Fanara et al. 2012), which indicates that PD patients have a reduced rate of microtubule-driven vesicle transport. CHGB has been utilized as a marker for regulated secretion of dense core vesicles and shown to be reduced by radiolabeled tracing of axonal transport dynamics (Constantinescu et al. 2010). As genetic ablation of CHGB in mice has been shown to reduce the monoamine content and secretion rate of large dense core vesicles (Diaz-Vera et al. 2010), it is possible that the reduced levels of CHGB in PD CSF can indicate a reduced function of dopamine transmission or be indicative of a general loss of dopamine neurons; in this regard, it would be instructive to measure CHGB levels in longitudinal samples from PD patients.

Although we do not detect a significant difference by univariate analysis in either CHGB peptide in ALS CSF (Table 2), in multivariate models, reduced levels of CHGB peptides contributed to separation of ALS.S from control (Fig. 4b). Genetic variants in CHGB (P413L) have previously been shown to impart an increased risk for ALS (Gros-Louis et al. 2009; Ohta et al. 2016). This variant has been shown to bind to and alter secretion of mutated but not wild-type SOD1 (Urushitani et al. 2006). In this regard, it should be noted that the current study has focused on sporadic non-mutated SOD1 ALS cases; however, misfolded SOD-1 aggregates are generally present in sporadic non-SOD1 ALS patient’s glial and neuronal cells in the spinal cord (Forsberg et al. 2010, 2011, 2019).

We have also detected reductions in UCHL1 (PARK5) in PD CSF. Reduced levels of UCHL1 in PD CSF have previously been demonstrated by ELISA methods (Mondello et al. 2014). PD-associated variants in the gene encoding alpha-synuclein (α-SYN) inhibit the secretion of UCHL1 (Konya et al. 2011); the PD-associated serine protease htrA2 has been shown to cleave UCHL1 (Park et al. 2011); and UCHL1 was identified as interacting with the Parkin E3 ubiquitin ligase in double affinity proteomics (Davison et al. 2009) and is a substrate for Parkin (McKeon et al. 2015). Furthermore, UCHL1 has been shown to interact with membranes following farnesylation—which also increases the level of UCHL1 interaction with α-SYN (Liu et al. 2009). Mice deleted for lipoprotein lipase show altered α-SYN and uchl1 expression and increased α-SYN ubiquitination and aggregation, suggesting a link between fatty acid metabolism and UCHL1 deubiquitination of α-SYN (Yang et al. 2015a). UCHL1 has also been shown to destabilize mTOR complex 1 by antagonizing the ubiquitination of raptor (Yang et al. 2015a). Analysis of UCHL1 in CSF supports the idea that this protein may have diagnostic value in multivariate models of PD but needs further validation in additional cohorts (Dos Santos et al. 2018).

There is general indication that utilizing two peptide standards from a single protein gives more reliable detection of alterations in protein levels. This was clearly the case in this study for CHGB (GEA and NYL), where PD patients had 68.0% and ALS 81% and 77% of the level of control CSF. This validates that these two peptides come from the same transcript or isoform of CHGB and perform similarly well in MRM assays (correlation 0.97 across all samples, not shown). Conversely, while two peptides from UCHL1 (LGF and LGV) are both significantly different in ALS compared to control, they exhibit lower correlation (0.73 across all samples) within individual samples. These peptides are measured near the detection level of the instrument and required manual quantification of individual peaks, which was more robust for the LGV peptide (Supplementary Table 2). They are altered differently, 122 and 184% respectively in ALS and 83 and 81% in PD. In fact, only the UCHL1 LGF peptide is significantly different in PD compared to control (*p* value 0.003), which indicates less reliability of these measurements as a candidate biomarker for PD.

Another peptide pair that diverged was CTSD (VST and YSQ), where only VST was significantly increased in ALS (113% of control, *p* value 0.009). This difference was driven primarily by a single ALS sample in which YSQ was undetectable above the noise level, indicating complete absence of this peptide. This is likely due to homozygosity for a known genetic variant encoding a V58A change within the CTSD protein. While this genetic variant has been shown to increase the risk of developing Alzheimer’s disease (Schuur et al. 2011), it was not associated with increased risk of ALS or PD in a Han Chinese population (Xi et al. 2018). Due to the apparent detection of variant alleles by MRM, only the VST peptide of CTSD was used for analysis and modeling. We also detected a number of patients with approximately 50% of the average level of peptide, suggesting genetic heterozygosity at this allele.

CTSD is a key lysosomal protease involved in degradation of alpha-synuclein that is also implicated in the lysosomal storage neurodegenerative disorder Ceroid lipofuscinosis, neuronal, type 10 (Siintola et al. 2006). An increase in mRNA encoding CTSD has previously been detected in post-mortem spinal cord of ALS patients (Offen et al. 2009), but no previous measurements of CTSD in CSF have been correlated to ALS. Here, we report an increase in ALS patients compared to control which is more pronounced in short-lived patients, but not significantly different between short- and long-lived patients.

UCHL1 is highly abundant in the brain (Bishop et al. 2016), and any studies have used UCHL1 (PGP9.5) immunohistochemistry or synthetic genetic reporter constructs to label cutaneous sensory and motor neurons due to high levels of basal expression of uchl1 in these cells. But, while this marker has been used to study mouse models of ALS (Genc et al. 2015; Gautam et al. 2016) and to investigate epidermal small-fiber neuropathy in ALS (Weis et al. 2011), we report the first use of peptide quantification in CSF to evaluate ALS levels of UCHL1. The increased CSF levels of UCHL1 in patients with ALS may be a marker for increased cellular degeneration and loss of UCHL1 to the extracellular environment similar to models proposed for increased Neurofilament light (NfL) in ALS CSF (Bridel et al. 2019). Increases in UCHL1 have been detected using proteomic analysis of skin fibroblast cultures from patients with spinal muscular atrophy (SMA), a lethal hereditary condition caused by mutations in the survival of motor neuron 1 (SMN) gene (Hsu et al. 2010). But, while UCHL1 was shown to directly interact with and increase ubiquitination of SMN and is increased in a mouse model of SMA, it is instructive to note that pharmacological inhibition of UCHL1 exacerbates the disease in the mouse model (Powis et al. 2014). This suggests that UCHL1 may not be a good target for therapeutic repression, which is supported by the clear deficits in the corticospinal motor neurons in mice deleted for the *uchl1* gene (Jara et al. 2015). Together, this suggests that UCHL1 may be a useful biomarker for either neuronal integrity, or improved ubiquitin-proteasomal function following therapeutic remediation at another molecular target.

GPNMB (glycoprotein nonmetastatic melanoma protein B) (also called osteoactivin) is a single-pass transmembrane protein that in upregulated by both lysosomal stress and inhibition of mTORC1 (Gabriel et al. 2014). GPNMB is a regulator of melanosome formation and protein degradation pathways (van der Lienden et al. 2018) and inhibits inflammation (Neal et al. 2018) and amyloid formation (Yang et al. 2018). Increases in GPNMB mRNA were measured in lumbar spinal cord of SOD1^G93A^ mice and GPNMB protein was increased in CSF of sporadic ALS (Tanaka et al. 2012). GPNMB binds to the SOD1^G93A^ variant and modulates the muscular and neurodegenerative actions of that molecule; and co-expression of GPNMB or direct injection into gastrocnemius muscle alleviates muscle degeneration in a background of SOD1^G93A^ mice (Nagahara et al. 2015).

In this study, we detected a significant increase in GPNMB protein in CSF of ALS patients that was more pronounced in shorter-lived ALS patients. It is interesting to note that both GPNMB and UCHL1 levels are increased by inhibition of mTORC1 signaling; in the current study, there is a relatively high correlation between GPNMB and UCHL1-LGF (0.49). The tryptic peptide we are employing to measure GPNMB levels is from the extracellular domain, which means that we may be measuring a cleaved protein fragment in the CSF (Rose et al. 2010). Quantification of additional tryptic peptides will allow more detailed analysis of which segments of GPNMB are increased in CSF of short-lived ALS patients.

Genetic variants within the GPNMB gene are associated with age of onset of PD (Iwaki et al. 2019) and expression is increased in the brains of patients with PD (Moloney et al. 2018). gpnmb mRNA is increased in mouse models of, and patients with, the lysosomal lipid storage disorder Niemann-Pick Type C (Marques et al. 2016). GPNMB has also been identified as a biomarker in CSF for neuropathic forms of Gaucher’s disease (types 2 and 3) (Zigdon et al. 2015) that are caused by mutations in GBA, a Parkinson’s disease-associated gene (Siebert et al. 2014). While we did not detect statistically significant changes in levels of GPNMB in PD patients in this cohort, it will be informative to measure these levels in PD patients with a GBA variant known to increase risk of developing PD or in another cohort. While we did not detect any significant changes in the two GBA peptides in PD patients compared to control, these peptides were measured at the limit of detection and may be better quantified using a slightly more sensitive analytical system.

### Longevity Biomarkers in ALS

The average survival of ALS patients from first diagnosis to death is approximately 3 years, with about 10% percent of patients surviving for more than 10 years (Forsgren et al. 1983). In this study, we have chosen to study a population randomly selected without regard to longevity from the Umeå University, Department of Neurology ALS biobank in which, by coincidence, one half of the cohort died within 1 year of CSF collection and the other half survived for at least 500 days post-sampling. While there are currently no robust clinical or diagnostic markers for prognosis of survival time, such tools would greatly aid in treatment and development of therapeutics (Bakkar et al. 2015). Several recent studies have sought to identify fluid biomarkers for longevity prognosis in ALS, including identification of neurofilament light chain (NfL) in CSF and plasma as significantly higher in ALS, and correlated to shorter survival (Lu et al. 2015); basic fibroblast growth factor 2 (bFGF) increased in ALS with higher levels correlating to longer survival (Gong et al. 2015); low creatine kinase association with longer survival (Gibson et al. 2015); and increased levels of macrophage inflammatory protein-1 alpha (MIP-1α) in both CSF and serum with an inverse correlation to progression of disease (Yang et al. 2015b). In the current study, we have detected increases in GPNMB and UCHL1 in ALS patients, which are driven by the shorter-lived patients; no significant change was detected between longer-lived ALS patients and control CSF. Additionally, we detect a significant difference between the short- and long-lived ALS patients for both proteins. This indicates that UCHL1 and GPNMB are good candidate biomarkers for longevity in ALS; multivariate modeling and ROC analysis indicate that these proteins can separate patients based on survival time and may be useful to predict ALS longevity. While CTSD is altered in the ALS cohort compared to control, we detect no difference in levels of this protein between short- and long-lived patients. This suggests that CTSD represents a common molecular pathology amongst both short- and long-lived ALS patients, while increases in UCHL1 and GPNMB in short-lived represent more aggressive molecular etiologies. In this case, it is easy to visualize the detrimental effect that grouping of these distinct molecular etiologies into a single ALS cohort might have upon untargeted clinical trials absent stratifying biomarkers.

### Metabolite Levels Support Distinct Longevity Prediction in the ALS Subgroups Defined Here

Despite the very small number of samples in the short- and long-lived ALS groups, we feel that it is important to report these initial experiments due to the significance such a differential diagnostic could have on drug development. So, to support the proteomics findings, we have compared for the first time here metabolomics measurements of the two groups. Analysis of the previously published metabolomics datasets reveals that long-lived ALS patients exhibit greater increases in CSF levels of alanine, creatine, propyleneglycol and the branched-chain amino acids isoleucine, valine, and leucine compared to controls than short-lived ALS patients. Conversely, short-lived ALS patients have lower creatinine, myoinositol, and 5-carbon sugars and their derivatives in CSF compared to control.

## Conclusion

We have attempted to cover a range of cellular pathways that may impact proteostasis, including vesicle trafficking, lysosomal targeting, and ubiquitin-proteasomal and protease degradation systems. We have detected distinct alterations in both Parkinson’s disease and ALS compared to control CSF. In PD, we detect alterations in dense-core vesicle, lysosome, and ubiquitin-proteasome regulation. For ALS, we observe distinct patterns of alterations in protein homeostasis pathways between short- and long-lived patients and identify candidate biomarkers for patient stratification into clinical trials.

## Electronic Supplementary Material

ESM 1 (DOCX 404 kb)

## Abbreviations

α-syn: Alpha-synuclein
ALS: Amyotrophic lateral sclerosis
ALS.L: Long-lived ALS
ALS.S: Short-lived ALS
ANOVA: Analysis of variance
AUC: Area under the curve
CV: Coefficient of variation
CE: Collision energy
CHGB: Chromogranin B/secretogranin-1
CTSD: Cathepsin D
GBA: Glucosylceramidase
GPNMB: Transmembrane glycoprotein NMB
LC-MS: Liquid chromatography-mass spectrometry
MRM: Multiple reaction monitoring
NAT: Native peptide
NMR: Nuclear magnetic resonance spectroscopy
OPLS-DA: Orthogonal projection to latent structures discriminant analysis
PCA: Principal component analysis
ROC: Receiver operating characteristic
SIS: Stable isotope-labeled standard
SOD1: Superoxide dismutase 1
UCHL1: Ubiquitin carboxyl-terminal hydrolase isozyme L1
UK PDSBB: United Kingdom Parkinson’s Disease Society Brain Bank
