# Supplementary material for: Targeted Multiple Reaction Monitoring Analysis of CSF Identifies UCHL1 and GPNMB as Candidate Biomarkers for ALS
Source: J Mol Neurosci. 2019 Nov 12;69(4):643–57. doi: 10.1007/s12031-019-01411-y (PMC6858390; doi:10.1007/s12031-019-01411-y)
Supplement: Supplementary file 1 — (DOCX 404 kb) [file 12031_2019_1411_MOESM1_ESM.docx]

Supplementary Table 1. Sequences and optimized transitions of target peptides.

Transitions for quantification are in **bold**.

| Peptides | NAT/  SIS | Sequence | Q1 | Q3 | Ion Name | CE | RT (min) |
| --- | --- | --- | --- | --- | --- | --- | --- |
| CHGB-GEA | NAT | GEAGAPGEEDIQGPTK | **778.4** | **1170.6** | **y11** | **25.1** | **3.3** |
|  |  |  | 778.4 | 685.3 | y14 | 25.1 | 3.3 |
|  |  |  | 778.4 | 621.3 | y12 | 25.1 | 3.3 |
|  | SIS | GEAGA-[U-13C5,15N-Pro]-GEEDIQGPTK-acid | **781.4** | **1176.6** | **y11** | **25.1** | **3.3** |
|  |  |  | 781.4 | 688.3 | y14 | 25.1 | 3.3 |
|  |  |  | 781.4 | 624.3 | y12 | 25.1 | 3.3 |
| CHGB-NYL | NAT | NYLNYGEEGAPGK | 706.3 | 1134.5 | y11 | 22.9 | 6.8 |
|  |  |  | **706.3** | **1021.5** | **y10** | **22.9** | **6.8** |
|  |  |  | 706.3 | 744.4 | y8 | 22.9 | 6.8 |
|  | SIS | NYLNYGEEGA-[U-13C5,15N-Pro]-GK-acid | 709.3 | 1140.6 | y11 | 22.9 | 6.8 |
|  |  |  | **709.3** | **1027.5** | **y10** | **22.9** | **6.8** |
|  |  |  | 709.3 | 750.4 | y8 | 22.9 | 6.8 |
| CTSD-VST | NAT | VSTLPAITLK | 521.8 | 943.6 | y9 | 17.2 | 14.2 |
|  |  |  | 521.8 | 856.6 | y8 | 17.2 | 14.2 |
|  |  |  | **521.8** | **642.4** | **y6** | **17.2** | **14.2** |
|  | SIS | [U-13C5,15N-Val]-STLPAITLK-acid | 524.8 | 943.6 | y9 | 17.2 | 14.2 |
|  |  |  | 524.8 | 856.6 | y8 | 17.2 | 14.2 |
|  |  |  | **524.8** | **642.4** | **y6** | **17.2** | **14.2** |
| CTSD-YSQ | NAT | YSQAVPAVTEGPIPEVLK | **949.5** | **1349.8** | **y13** | **30.4** | **16.4** |
|  |  |  | 949.5 | 1082.6 | y10 | 30.4 | 16.4 |
|  |  |  | 949.5 | 852.5 | y8 | 30.4 | 16.4 |
|  | SIS | YSQA-[U-13C5,15N-Val]-PAVTEGPIPEVLK-acid | **952.5** | **1349.8** | **y13** | **30.4** | **16.4** |
|  |  |  | 952.5 | 1082.6 | y10 | 30.4 | 16.4 |
|  |  |  | 952.5 | 852.5 | y8 | 30.4 | 16.4 |
| DJ1-EGP | NAT | EGPYDVVVLPGGNLGAQNLSESAAVK | **862.1** | **863.5** | **y18** | **26.2** | **17.6** |
|  |  |  | 862.1 | 806.9 | y17 | 26.2 | 17.6 |
|  |  |  | 862.1 | 972.5 | b9 | 26.2 | 17.6 |
|  | SIS | EGPYD-V-V-V-[U-13C6,15N-Leu]-PGGNLGAQNLSESAAVK-acid | **864.5** | **867** | **y18** | **26.2** | **17.6** |
|  |  |  | 864.5 | 806.9 | y17 | 26.2 | 17.6 |
|  |  |  | 864.5 | 979.5 | b9 | 26.2 | 17.6 |
| DJ1-GPG | NAT | GPGTSFEFALAIVEALNGK | **641** | **730.4** | **y7** | **18.3** | **23.3** |
|  |  |  | 641 | 631.3 | y6 | 18.3 | 23.3 |
|  |  |  | 641 | 802.4 | b16 | 18.3 | 23.3 |
|  | SIS | GPGTSFEFALAI-[U-13C5,15N-Val]-EALNGK-acid | **643** | **736.4** | **y7** | **18.3** | **23.3** |
|  |  |  | 643 | 631.3 | y6 | 18.3 | 23.3 |
|  |  |  | 643 | 805.4 | b16 | 18.3 | 23.3 |
| GBA-NFV | NAT | NFVDSPIIVDITK | 730.9 | 1199.7 | y11 | 23.7 | 18.3 |
|  |  |  | **730.9** | **1100.6** | **y10** | **23.7** | **18.3** |
|  |  |  | 730.9 | 898.6 | y8 | 23.7 | 18.3 |
|  | SIS | NFVDSPIIVDIT-(U-13C6, 15N2-Lys)-acid | 734.9 | 1207.7 | y11 | 23.7 | 18.3 |
|  |  |  | **734.9** | **1108.6** | **y10** | **23.7** | **18.3** |
|  |  |  | 734.9 | 906.6 | y8 | 23.7 | 18.3 |
| GBA-SYF | NAT | SYFSEEGIGYNIIR | 824.4 | 1250.6 | y11 | 26.6 | 16.1 |
|  |  |  | 824.4 | 905.5 | y8 | 26.6 | 16.1 |
|  |  |  | **824.4** | **735.4** | **y6** | **26.6** | **16.1** |
|  | SIS | SYFSEEGIGYNII-[U-13C6,15N4-Arg]-acid | 829.4 | 1260.6 | y11 | 26.6 | 16.1 |
|  |  |  | 829.4 | 915.5 | y8 | 26.6 | 16.1 |
|  |  |  | **829.4** | **745.4** | **y6** | **26.6** | **16.1** |
| GPNMB-AYV | NAT | AYVPIAQVK | **494.8** | **754.5** | **y7** | **16** | **8.1** |
|  |  |  | 494.8 | 655.4 | y6 | 16 | 8.1 |
|  |  |  | 494.8 | 558.4 | y5 | 16 | 8.1 |
|  | SIS | AY-[U13C5,15N-Val]-PIAQVK-acid | **497.8** | **760.5** | **y7** | **16** | **8.1** |
|  |  |  | 497.8 | 655.4 | y6 | 16 | 8.1 |
|  |  |  | 497.8 | 558.6 | y5 | 16 | 8.1 |
| UCHL1-LGF | NAT | LGFEDGSVLK | **532.8** | **951.5** | **y9** | **17.5** | **12.4** |
|  |  |  | 532.8 | 894.5 | y8 | 17.5 | 12.4 |
|  |  |  | 532.8 | 618.3 | y6 | 17.5 | 12.4 |
|  | SIS | LGFEDGS-[U-13C5,15N-Val]-LK-acid | **535.8** | **957.5** | **y9** | **17.5** | **12.4** |
|  |  |  | 535.8 | 900.5 | y8 | 17.5 | 12.4 |
|  |  |  | 535.8 | 624.4 | y6 | 17.5 | 12.4 |
| UCHL1-LGV | NAT | LGVAGQWR | **443.7** | **773.4** | **y7** | **14.8** | **7** |
|  |  |  | 443.7 | 617.3 | y5 | 14.8 | 7 |
|  |  |  | 443.7 | 546.3 | y4 | 14.8 | 7 |
|  | SIS | LG-[U-13C5,15N-Val]-AGQ-W-R-acid | **446.8** | **779.4** | **y7** | **14.8** | **7** |
|  |  |  | 446.8 | 617.3 | y5 | 14.8 | 7 |
|  |  |  | 446.8 | 546.3 | y4 | 14.8 | 7 |

Supplementary Table 2 Standard curves, LODs and LLOQs of peptide assays

| **Peptide** | **Protein MW** | **Formula*** | **R square** | **Median CV of standard curves^Δ^** | **LOD** | | **LLOQ** | |
| --- | --- | --- | --- | --- | --- | --- | --- | --- |
|  |  |  |  |  | fmol/μl | ng/ml | fmol/μl | ng/ml |
| **CHGB-GEA** | 78276 | y = 0.0861x – 0.001037 | R² = 0.996 | 3.10% | 0.0075 | 0.58 | 0.069 | 5.37 |
| **CHGB-NYL** | 78276 | y = 0.164x – 2.7e-5 | R² = 0.990 | 5.60% | 0.012 | 0.91 | 0.069 | 5.37 |
| **CTSD-VST** | 44552 | y = 0.499x + 0.000141 | R² = 0.991 | 1.30% | 0.0037 | 0.17 | 0.011 | 0.50 |
| **CTSD-YSQ** | 44552 | y = 1.609x - 0.00037 | R² = 0.987 | 11.00% | 0.013 | 0.59 | 0.069 | 1.78 |
| **DJ1-EGP** | 19891 | y = 2.6828x + 0.0156 | R² = 0.980 | 11.50% | 0.040 | 0.99 | 0.21 | 4.09 |
| **DJ1-GPG** | 19891 | y = 5.191x-0.217 | R² = 0.941 | 16.70% | 0.086 | 1.71 | 0.21 | 4.09 |
| **GBA-NFV** | 59716 | y = 27.78x + 0.2632 | R² = 0.902 | 23.60% | 0.013 | 0.75 | 0.038 | 2.24 |
| **GBA-SYF** | 59716 | y = 15.81x + 0.3444 | R² = 0.872 | 22.80% | 0.015 | 0.87 | 0.044 | 2.61 |
| **GPNMB-AYV** | 62643 | y = 8.202x + 0.0522 | R² = 0.980 | 5.20% | 0.0054 | 0.34 | 0.016 | 1.02 |
| **UCHL1-LGF** | 24824 | y = 45.70x -0.158 | R² = 0.954 | 12.70% | 0.0085 | 0.21 | 0.026 | 0.63 |
| **UCHL1-LGV** | 24824 | y = 23.07x – 0.1309 | R² = 0.952 | 8.50% | 0.0086 | 0.21 | 0.026 | 0.64 |

Notes: * x was spiked SIS, y was ratio of SIS response/NAT area. **^Δ^** the median of all CVs across all concentration levels of each standard curve. Peptides concentration in each sample was calculated by (y-intercept)/ (1- intercept)*C_IS_, C_IS_ was concentration of SIS spiked in individual samples.


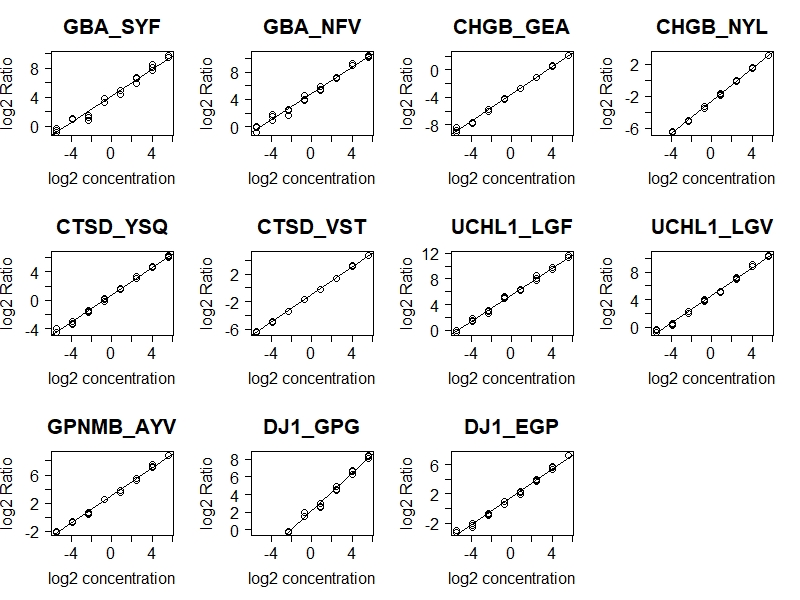


Supplementary Figure 1. Standard curves of 11 peptide assays in pooled CSF digests. Y-axis is the log2 transformed response ratio of SIS to NAT and X-axis is the log2 transformed SIS concentration.


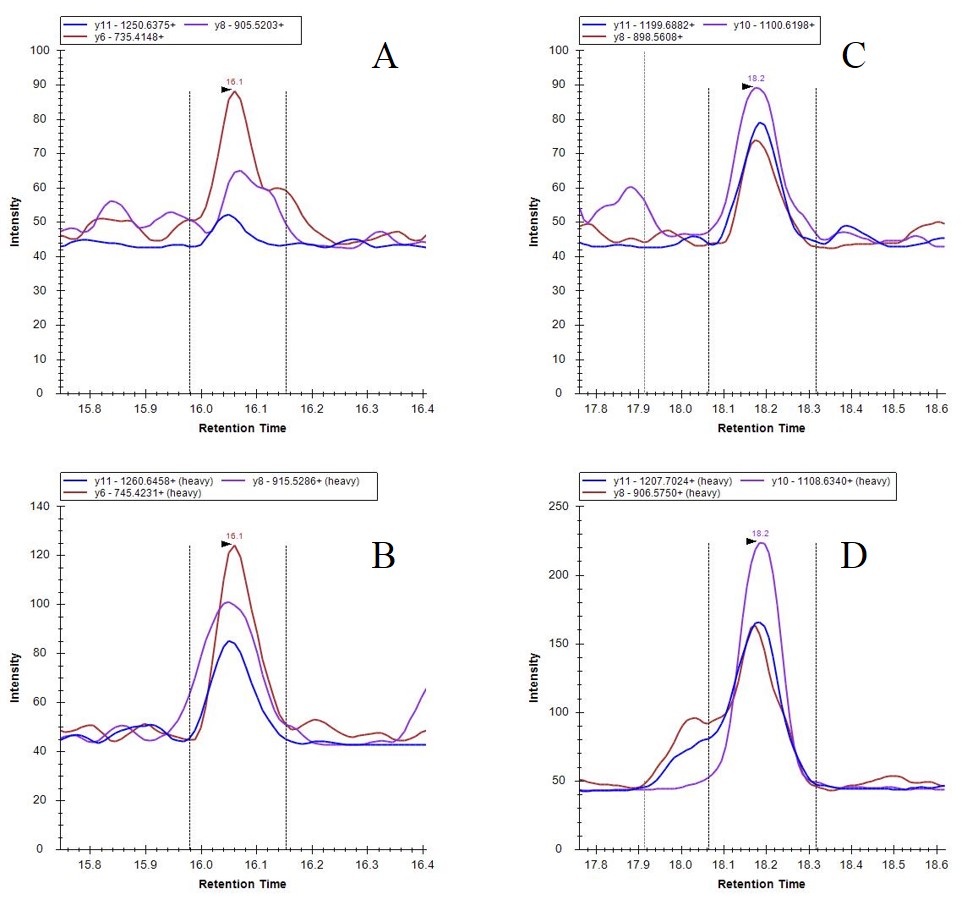


Supplementary Figure 2. GBA-SYF and GBA-NFV in sample C39. A, B: NAT and SIS of GBA-SYF; C, D: NAT and SIS of GBA-NFV. Levels of both SISs were spiked in at 0.1fmol/μl

Supplemental Table 3. Run order based on constrained randomization.

| Runorder | Sample | Match | Group | Sex |
| --- | --- | --- | --- | --- |
| 1 | C57 | 6 | PD | m |
| 2 | C39 | 6 | ALS | m |
| 3 | C16 | 6 | Control | m |
| 4 | C03 | 9 | Control | f |
| 5 | C35 | 9 | ALS | f |
| 6 | C61 | 9 | PD | f |
| 7 | C67 | 15 | PD | m |
| 8 | C28 | 15 | ALS | m |
| 9 | C42 | 15 | Control | m |
| 10 | C34 | 21 | ALS | m |
| 11 | C54 | 21 | PD | m |
| 12 | C31 | 21 | Control | m |
| 13 | C18 | 8 | ALS | m |
| 14 | C14 | 8 | Control | m |
| 15 | C48 | 8 | PD | m |
| 16 | C25 | 8 | Control | m |
| 17 | C22 | 16 | ALS | m |
| 18 | C11 | 16 | Control | m |
| 19 | C68 | 16 | PD | m |
| 20 | C13 | 19 | Control | m |
| 21 | C62 | 19 | PD | m |
| 22 | C04 | 11 | Control | f |
| 23 | C09 | 11 | ALS | f |
| 24 | C47 | 11 | PD | f |
| 25 | C56 | 4 | PD | m |
| 26 | C43 | 4 | Control | m |
| 27 | C46 | 4 | ALS | m |
| 28 | C49 | 3 | PD | f |
| 29 | C01 | 3 | Control | f |
| 30 | C10 | 3 | ALS | f |
| 31 | C58 | 12 | PD | f |
| 32 | C23 | 12 | ALS | f |
| 33 | C30 | 12 | Control | f |
| 38 | C53 | 2 | PD | f |
| 39 | C08 | 2 | ALS | f |
| 34 | C51 | 18 | PD | m |
| 35 | C06 | 18 | Control | m |
| 36 | C41 | 18 | Control | m |
| 37 | C21 | 18 | ALS | m |
| 40 | C05 | 14 | ALS | m |
| 41 | C59 | 14 | PD | m |
| 42 | C45 | 14 | Control | m |
| 43 | C32 | 14 | Control | m |
| 44 | C64 | 7 | PD | m |
| 45 | C15 | 7 | Control | m |
| 46 | C37 | 7 | ALS | m |
| 47 | C29 | 20 | Control | m |
| 48 | C20 | 20 | ALS | m |
| 49 | C65 | 20 | PD | m |
| 50 | C66 | 1 | PD | f |
| 51 | C27 | 1 | Control | f |
| 52 | C07 | 1 | Control | f |
| 53 | C38 | 1 | ALS | m |
| 54 | C17 | 5 | ALS | m |
| 55 | C50 | 5 | PD | m |
| 56 | C33 | 5 | Control | m |
| 57 | C40 | 17 | ALS | m |
| 58 | C63 | 17 | PD | m |
| 59 | C12 | 17 | Control | m |
| 60 | C55 | 10 | PD | f |
| 61 | C02 | 10 | Control | f |
| 62 | C36 | 10 | ALS | f |
| 63 | C24 | 13 | Control | m |
| 64 | C19 | 13 | ALS | m |
| 65 | C60 | 13 | PD | m |
| 66 | C44 | 22 | Control | m |
| 67 | C26 | 22 | ALS | m |

**Uploaded data files for Skyline:**

**Data link:**

<https://panoramaweb.org/labkey/targetedms/Tutorials/zhu_tutorials/PD%20ALS%20candidates%20MRM/showList.view>?

username: shaochunzhu@gmail.com

pw: UmeatargetProteomics

**1. Three files for standard curves:**

20151108SC all transitions_2016-12-24_09-27-22.sky.zip

20151108SC2 all transitions_2016-12-24_09-28-08.sky.zip

20151108SC4 all transitions_2016-12-24_09-28-53.sky.zip

**2. Five files for samples measurement:**

20151108 sample 11 13 14 18 22 25 31 34 42 48 54 68 all transitions_2016-12-24_09-21-21.sky.zip

20151108 sample 01 04 09 10 23 43 46 47 49 56 58 62 all transitions_2016-12-24_09-23-40.sky.zip

20151108 sample 05 06 08 21 30 32 41 45 51 53 59 64 all transitions_2016-12-24_09-24-51.sky.zip

20151108 sample 07 15 17 20 27 29 33 37 38 50 65 66 all transitions_2016-12-24_09-25-50.sky.zip

20151108 sample 02 12 19 24 26 36 40 44 55 60 63 all transitions_2016-12-24_09-26-42.sky.zip

**3. Transitions for quantification listed in Supplemental Table 1**
